# A Three-Dimensional Modeling Approach for Carbon Nanotubes Filled Polymers Utilizing the Modified Nearest Neighbor Algorithm

**DOI:** 10.3390/polym16192824

**Published:** 2024-10-06

**Authors:** Junpu Wang, Xiaozhuang Yue, Yuxuan Wang, Liupeng Di, Wenzhi Wang, Jingchao Wei, Fei Yu

**Affiliations:** 1 College of Mechanical and Electrical Engineering, Shaanxi University of Science and Technology, Xi’an 710021, China; 220512097@sust.edu.cn (X.Y.); wyuxuan2000@163.com (Y.W.); 230512091@sust.edu.cn (L.D.); 2 School of Aeronautics, Northwestern Polytechnical University, Xi’an 710072, China; wangwenzhi@nwpu.edu.cn; 3 National Key Laboratory of Strength and Structural Integrity, Aircraft Strength Research Institute of China, Xi’an 710065, China; weijingchao66@aliyun.com (J.W.); yuf078@avic.com (F.Y.)

**Keywords:** carbon nanotube, epoxy, representative volume element, finite element analysis, mechanical property

## Abstract

Carbon nanotubes (CNTs) are extensively utilized in the fabrication of high-performance composites due to their exceptional mechanical, electrical, and thermal characteristics. To investigate the mechanical properties of CNTs filled polymers accurately and effectively, a 3D modeling approach that incorporates the microstructural attributes of CNTs was introduced. Initially, a representative volume element model was constructed utilizing the modified nearest neighbor algorithm. During the modeling phase, a corresponding interference judgment method was suggested, taking into account the potential positional relationships among the CNTs. Subsequently, stress–strain curves of the model under various loading conditions were derived through finite element analysis employing the volume averaging technique. To validate the efficacy of the modeling approach, the stress within a CNT/epoxy resin composite with varying volume fractions under different axial strains was computed. The resulting stress–strain curves were in good agreement with experimental data from the existing literature. Hence, the modeling method proposed in this study provides a more precise representation of the random distribution of CNTs in the matrix. Furthermore, it is applicable to a broader range of aspect ratios, thereby enabling the CNT simulation model to more closely align with real-world models.

## 1. Introduction

Carbon nanotubes (CNTs) filled polymer composites find extensive applications in aerospace [1], the automotive industry [2], energy storage components [3], sensors [4], wind turbine blades [5], and other sectors due to their exceptional mechanical [6], thermal [7], and electrical properties [8]. The mechanical properties of CNTs filled polymers can be affected by various factors, including the volume fraction of the filler, aspect ratio, and curing temperature and time [9,10]. Experimental methods for studying carbon-based filled polymers typically require significant time and costs. For instance, preparing silica CNTs involves a number of intricate and time-intensive processes, including high-temperature drying and ultrasonic mixing [11,12]. In contrast, numerical simulations offer a more convenient and cost-effective approach for parametric research, with the finite element (FE) method being utilized for analyzing and solving mechanical, thermal, and electrical issues in carbon-based filled polymers.

To replicate the microstructure of CNTs filled polymers, representative volume elements (RVE) with a random distribution of CNTs are created using the random sequence adsorption (RSA) algorithms. For instance, CNTs are defined as one-dimensional rod elements, and a randomly distributed CNT network is generated based on the volume fraction [13]. Similarly, Matus et al. [14,15] developed an RVE model to predict the multi-axial strain-sensing response of CNTs filled polymers, where CNTs are randomly distributed as one-dimensional rod elements. While this simplistic and efficient modeling approach of CNTs can save modeling time and reduce complexity, the freedom of the rod elements is somewhat restricted. Furthermore, due to the differing degrees of freedom between the rod elements and three-dimensional (3D) elements, specific adjustments are necessary in the simulation. Consequently, an enhanced 3D statistical conductive network RVE model was formulated to investigate the electrically conductive characteristics of CNTs filled polymers [16,17]. In this model, the randomly distributed CNTs were simplified as “soft core” cylinders that are allowed to intersect. Similarly, in reference [18], a 3D statistical resistance network model was created to simulate the CNTs filled polymers. The carbon nanoparticles and CNT fillers were considered as solid spherical conductors and cylindrical conductors with a head, respectively. Both types of conductors were modeled as soft core structures, enabling penetration within the non-conductive polymer matrix. While this soft core model is highly efficient and can achieve high volume fractions, the fillers may intersect and overlap, deviating from the actual distribution of conductive fillers in the matrix. Subsequently, Wang et al. [19] introduced CNTs depicted as solid cylinders with hemispherical end caps. The position and orientation of CNTs were determined by midpoint coordinates, azimuth angles, and polar angles. Additionally, the hard-core RVE model was utilized to simulate CNTs filled composites across various volume fractions, with the CNTs defined by generating the two endpoint coordinates of each cylinder using the RSA algorithm [20,21]. Although the model generated by the RSA algorithm can achieve a random layout and strong discreteness of CNTs, it tends to reach saturation after a certain number of CNTs, making it challenging to introduce new CNTs and resulting in low space utilization efficiency. The calculation of new CNT positions is time-consuming, leading to reduced efficiency. Moreover, the aspect ratio of conventional CNTs typically ranges from 100 to 5000 [22], which significantly affects their mechanical, electrical, thermal, and other properties. For example, the ultra-long CNTs synthesized by Fei Wei’s research group possess a diameter of 2–6 nm and can attain lengths of up to 550 mm, yielding an aspect ratio that exceeds 100,000 [23]. However, in most of the literature, the aspect ratio of CNTs in the RVE models is limited to 1–50 [24,25,26], introducing a certain deviation from the actual model and real-world conditions.

**Figure 1:**
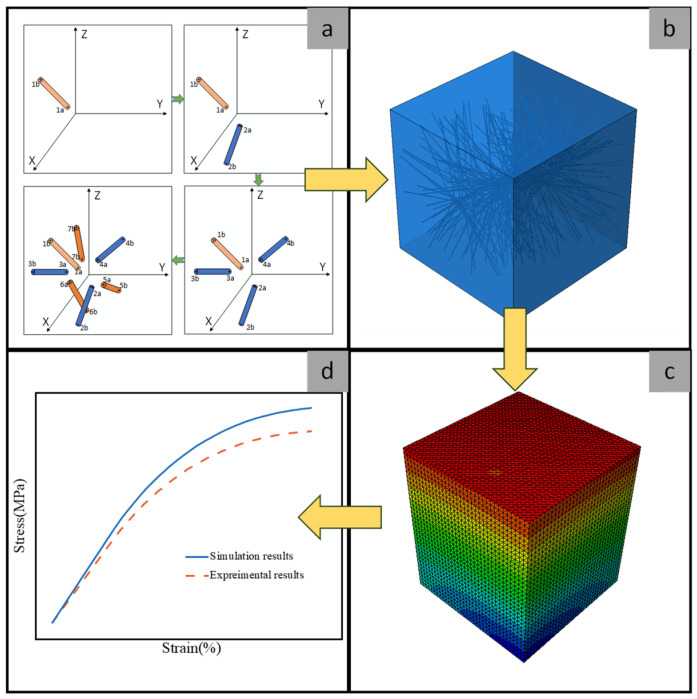
A schematic representation of the multi-scale analysis method: (**a**) generation of the CNTs; (**b**) establishment of the RVE model; (**c**) FE analysis; (**d**) comparison of the stress–strain curves.

To enhance the randomness and aspect ratio of CNTs generated within the RVE model, thereby aligning their microstructure more closely with the actual distribution and broadening the applicability of the RVE model, this study proposes a microscopic modeling approach for CNTs filled polymers. The objective is to establish an RVE model with a CNT aspect ratio far exceeding 50, and to simulate its mechanical properties through a multi-scale analysis. Initially, an RVE model that accurately represents the CNTs filled polymers is established based on the modified nearest distance algorithm [27]. Subsequently, FE analysis is conducted using the commercial software Abaqus 2022 to determine the stress and strain distributions. The stress–strain curve of the composite material is then derived through the volume averaging method. To validate the efficacy of the proposed method, the uniaxial tensile behavior of CNTs/epoxy resin under different displacement is simulated and compared with experimental data from the reference [28]. The schematic representation of the multi-scale analysis method is illustrated in Figure 1.

## 2. Establishment of the CNTs Filled Polymers Using the RVE Model

To effectively model the random distribution of CNTs within a polymer matrix, a 3D RVE modeling approach was created to simulate the mechanical properties of CNTs filled polymers.

Currently, the majority of theoretical algorithms in stochastic modeling heavily depend on computers for the generation of a significant quantity of pseudo-random numbers. Commonly used algorithms in this context encompass RSA [29], random sequence expansion (RSE) [30], and the NNA (NNA) integrated with the statistical distribution pattern of empirical data [31]. Furthermore, building upon the strengths of RSE and NNA, a modified NNA algorithm capable of producing RVE models that better align with the internal microstructure characteristics of the composite was introduced [27]. Consequently, in order to precisely depict the internal microstructure characteristics of CNTs filled polymers, we introduce a methodology for creating 3D coordinates of CNTs based on the modified NNA algorithm.

### 2.1. Generation of the CNTs Coordinates

**Figure 2:**
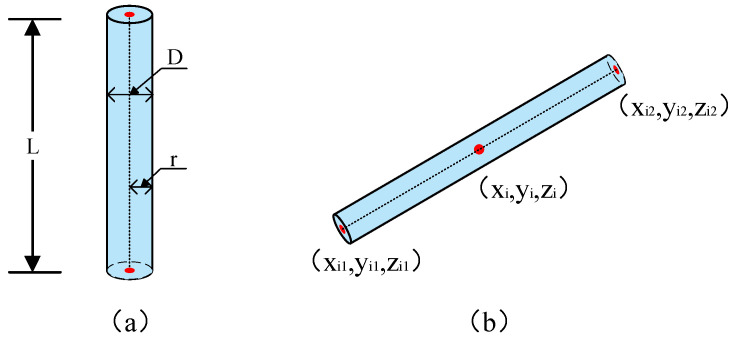
Modeling a 3D CNT: (**a**) defining a CNT and (**b**) configuring its position and orientation.

Due to the fact that the outermost layer of CNTs carries and transmits the primary external load, CNTs can be considered as cylindrical models [32]. In order to enhance computational efficiency, this study disregards the agglomeration and deformation of CNTs. Consequently, CNTs are treated as solid cylinders with a length of L and a radius of r (illustrated in Figure 2), and the following assumptions are made.

Firstly, the CNTs are considered as cylinders uniformly dispersed within the matrix, with their spatial coordinates and radii randomly distributed. To accommodate variations in filler sizes, a random fluctuation of ±5% is applied to both the axial and radial dimensions of the CNTs. Secondly, it is ensured that the CNTs do not overlap or intersect with each other, requiring the use of a hard-core model in this study.

To begin, let us assume that the midpoint coordinates of the *i*-th and *j*-th CNTs axes are $\left(x_{i},y_{i},z_{i}\right)$ and $\left(x_{j},y_{j},z_{j}\right)$, respectively. The calculation formula based on the modified NNA algorithm is as follows:

(1) $$\{\begin{array}{l} x_{j}=x_{i}+d_{ij}\times \cos\theta \times \sin\varphi \\ y_{j}=y_{i}+d_{ij}\times \cos\theta \times \cos\varphi \\ z_{j}=z_{i}+d_{ij}\times \sin\theta \end{array}$$

The distance between the midpoints of the *i*-th and *j*-th CNTs is denoted as *d_ij_*. The parameters *θ* and φ, which represent the directions, are defined as:

(2) $$\{\begin{array}{l} d_{ij}=r\times 0.1\times rand+r_{i}+r_{j}\\ \theta =rand\times 2\times \pi \\ \varphi =rand\times 2\times \pi \end{array}$$

where *r* is the cross-sectional radius of the cylinder, and rand is a randomly generated number between 0 and 1. As illustrated in Figure 2b, the CNT’s position can be ascertained upon acquiring its midpoint coordinates; however, its orientation necessitates determination based on the central coordinates of the two ends of the CNT. For the *i*-th CNT, assuming the central coordinates of its two ends are denoted as $\left(x_{i1},y_{i1},z_{i1}\right)$ and $\left(x_{i2},y_{i2},z_{i2}\right)$, respectively, the calculation formulas are presented in Equations (3) and (4):

(3) $$\{\begin{array}{l} x_{i1}=x_{i}+\frac{L_{i}}{2}\times \nu \times \cos(\omega )\\ y_{i1}=y_{i}+\frac{L_{i}}{2}\times \nu \times \sin(\omega )\\ z_{i1}=z_{i}+\frac{L_{i}}{2}\times \mu \end{array}$$

(4) $$\left\{ \begin{array}{l} x_{i2}=2x_{i}-x_{i1}\\ y_{i2}=2y_{i}-y_{i1}\\ z_{i2}=2z_{i}-z_{i1} \end{array}\right.$$

where *L_i_* denotes the length of the *i*-th CNT. The parameters representing direction *μ*, *ν*, and *ω* are defined as follows:

(5) $$\{\begin{array}{l} \mu =1.0-2.0\times rand\\ \nu =\sqrt{1.0-\mu^{2}}\\ \omega =rand\times 2\times \pi \end{array}$$

### 2.2. Interference Judgment

In the 3D model derived from the algorithm described above, the possibility of contact between the two generated cylinders exists, which contradicts the assumption that CNTs cannot overlap or intersect. Hence, it becomes imperative to ascertain the positional correlation between the generated cylinders. In cases where contact is identified, the cylinder must be regenerated until it aligns with the specified criteria. The model and algorithms discussed in references [16,17,18,19] illustrate that the distance between two cylinders can be redefined as the distance between line segments. However, the minimum distance generated by this algorithm frequently does not correspond to the true value and fails to consistently represent the actual shortest distance. Consequently, certain CNTs that have not interacted or made contact may be inaccurately classified as interfering. To address this issue, the present article introduces an improved method based on the original algorithm to more accurately evaluate interference conditions. To further elucidate the distinctions between the proposed algorithm and those referenced in sources [16,17,18,19], we present a comprehensive comparison in Appendix A. This appendix encompasses a thorough analysis of scenarios in which the existing algorithms inadequately detect interference, accompanied by examples that illustrate how the enhanced algorithm introduced in this paper effectively resolves these challenges. Through these comparisons, the superiority of the new method becomes apparent, particularly in instances where the original approach erroneously classifies non-overlapping cylinders as overlapping.

**Figure 3:**
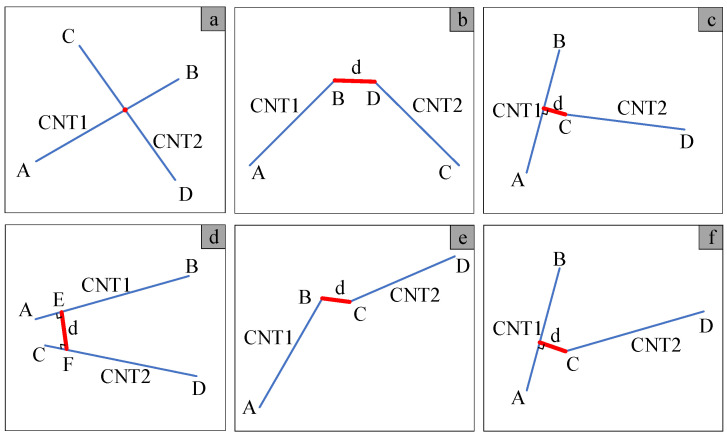
The positional relationship between two spatial line segments.

The potential positional configurations of two cylinders in three-dimensional space are illustrated in Figure 3. Given the coordinates of the four points A, B, C, and D in space as (*x*_11_, *y*_11_, *z*_11_), (*x*_12_, *y*_12_, *z*_12_), (*x*_21_, *y*_21_, *z*_21_), and (*x*_22_, *y*_22_, *z*_22_), the directional vectors of line segments ***AB*** and ***CD*** can be represented as:

(6) $$\begin{array}{l} AB=\left(x_{12}-x_{11},y_{12}-y_{11},z_{12}-z_{11}\right)\\ CD=\left(x_{22}-x_{21},y_{22}-y_{21},z_{22}-z_{21}\right) \end{array}$$

The positional relationship between line segments AB and CD can be categorized into two scenarios based on the value of ***m***, where ***m*** $=\left(AB\times CD\right)\cdot AC$. When m=0, ***AB*** and ***CD*** lie in the same plane; otherwise, they are not coplanar.

1. AB and CD are coplanar.

The intersection of two line segments can be ascertained through cross-disciplinary legislation, as articulated in the following manner:

(7) $$\{\begin{array}{l} a=\left(CA\cdot CD\right)\cdot \left(CB\cdot CD\right)\\ b=\left(AC\cdot AB\right)\cdot \left(AB\cdot AD\right) \end{array}$$

When a<0, and b<0, the segments AB and CD will be intersected, as illustrated in Figure 3a. This intersection implies an overlap of the CNTs, which contradicts the initial assumption. Therefore, it is imperative to generate new coordinates for the CNT.

When a≥0, or b≥0, the segments AB and CD will be not intersected. Scenarios illustrating the shortest distance between the two line segments are depicted in Figure 3b,c. To determine the shortest distance between the two line segments, it is essential to compute the point-to-point distance among the four endpoints A, B, C, and D, and to subsequently calculate the four point-to-line distances: endpoints C and D to segment AB, and A and B to segment CD. The minimum value among these eight distances represents the shortest distance between AB and CD.

2. AB and CD are noncoplanar.

Firstly, the perpendicular coordinates E and F of the common perpendicular of line segments AB and CD need to be determined [33]. Subsequently, the relationship between the common perpendicular point and the line segment should be estimated. For instance, considering point E and line segment AB, based on Equation (8), it is evident that it lies on line AB only when 0≤g≤1.

(8) $$g=\frac{AE\cdot AB}{\left|AB\right|}$$

When points E and F lie on the line segment (as depicted in Figure 3d), the shortest distance between ***AB*** and ***CD*** is equal to the length of the common perpendicular *d*.

(9) $$d=\frac{\left|AC\cdot \left(AB\times CD\right)\right|}{\left|AB\times CD\right|}$$

If points E and F do not lie entirely on a line segment, the shortest distance between the two line segments can be categorized into two situations: point-to-point distance (as illustrated in Figure 3e) and point-to-line distance (as depicted in Figure 3f). Likewise, the minimum distance among the eight distances is the shortest between AB and CD.

In summary, regardless of whether the two line segments are coplanar or noncoplanar, if the calculated shortest distance between them is below the specified threshold, it indicates an overlap between the two CNTs. Consequently, the newly formed cylinder needs to be eliminated and regenerated.

**Figure 4:**
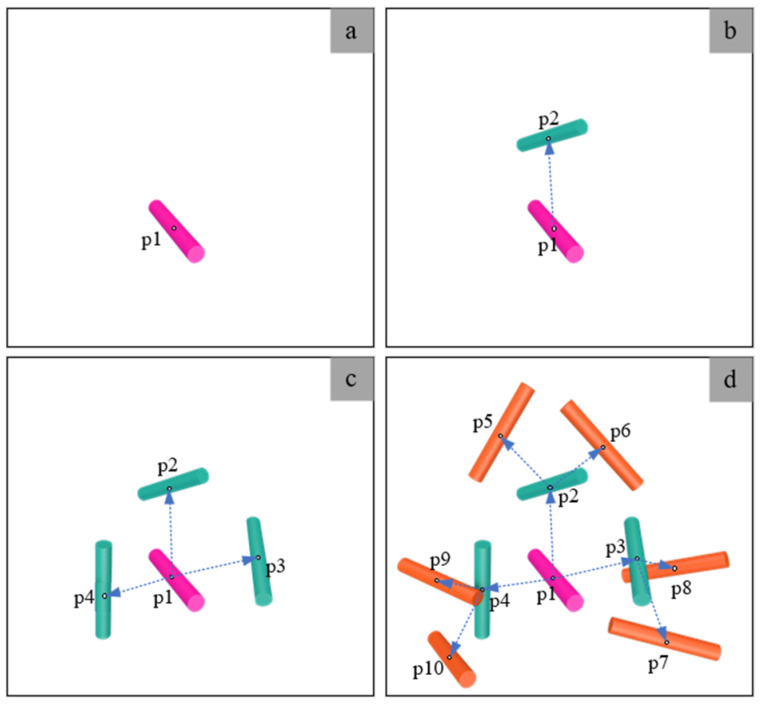
A schematic representation of the modified NNA algorithm.

Based on the modified NNA and the interference judgments outlined above, the procedure for creating an RVE model incorporating the CNTs is illustrated in Figure 4. Various colors in the figure denote the CNTs produced at different stages of the process. The generation sequence is detailed as follows:

- Randomly generate the 3D coordinates, length, and radius of the initial CNT within the spatial domain;
- Generate the spatial position coordinates, length, and radius of the second CNT using the modified NNA. Subsequently, assess the spatial relationship of the cylinder with the preceding one. In case of any interference, eliminate the newly generated cylinder and initiate the generation process again. Iterate this procedure until a non-interfering cylinder is produced. Finally, update the probability of generating the nearest distance;
- Increase the generation of additional cylinders in close proximity to the initial CNT;
- Repeat the preceding procedures iteratively until the desired volume fraction is attained.

### 2.3. Size of the RVE Model

The RVE model is an essential component in the analysis of non-uniform materials. To accurately depict macroscopic material behavior, the RVE model needs to encompass detailed microscopic characteristics of the composite material. For composites with identical filling volume fractions, it is evident that larger models incorporate more microstructural details. However, an excessively large model results in a higher number of meshes, escalating computational expenses. Hence, determining the optimal size of the RVE model is crucial for enhancing computational efficiency. Typically, the characteristic size of RVE models can be defined as the ratio of the edge length (*L_x_*) to the length of CNT *L*. To ensure the convergence of elastic properties, this ratio should be no less than 1.5 [34]. Previous studies have shown that satisfactory agreement between simulation and experimental outcomes is achieved when the ratio ranges from 2 to 8 [26]. Therefore, for a balance between accuracy and computational speed, an RVE model with a ratio of 2 is adequate for capturing the microstructural information of the CNTs filled polymers while maintaining a moderate quantity.

### 2.4. Boundary Conditions

The RVE model examines the macroscopic properties of composites by analyzing microstructure characteristics. To ensure deformation coordination, it is essential to apply periodic boundary conditions [35]. In the case of a 3D RVE model, the boundary conditions are established by translating the macroscopic strain field into microscopic displacements of boundary nodes [36]. By meeting the stress continuity condition, periodic boundary conditions in the X, Y, and Z directions can be implemented to fulfill the displacement continuity condition. Throughout the loading process, the nodes at the two corresponding edge interfaces in the model maintain a one-to-one correspondence, ensuring consistent deformation of the corresponding nodes. Consequently, the corresponding periodic boundary conditions can be represented in vector form as:

(10) $$\left\{ \begin{array}{l} u_{\left(x_{0},y,z\right)}=u_{\left(x_{1},y,z\right)}+u_{x}\\ u_{\left(x,y_{0},z\right)}=u_{\left(x,y_{1},z\right)}+u_{y}\\ u_{\left(x,y,z_{0}\right)}=u_{\left(x,y,z_{1}\right)}+u_{z} \end{array}\right.$$

where (*x*, *y*, *z*_0_) and (*x*, *y*, *z*_1_) represent the points located on the respective surface at the lower and upper ends of the model. The displacement vectors in the X, Y, and Z directions, denoted by $u_{x}$, $u_{y}$, $u_{z}$, are influenced by the loading conditions and the association between the node and the loading position.

**Figure 5:**
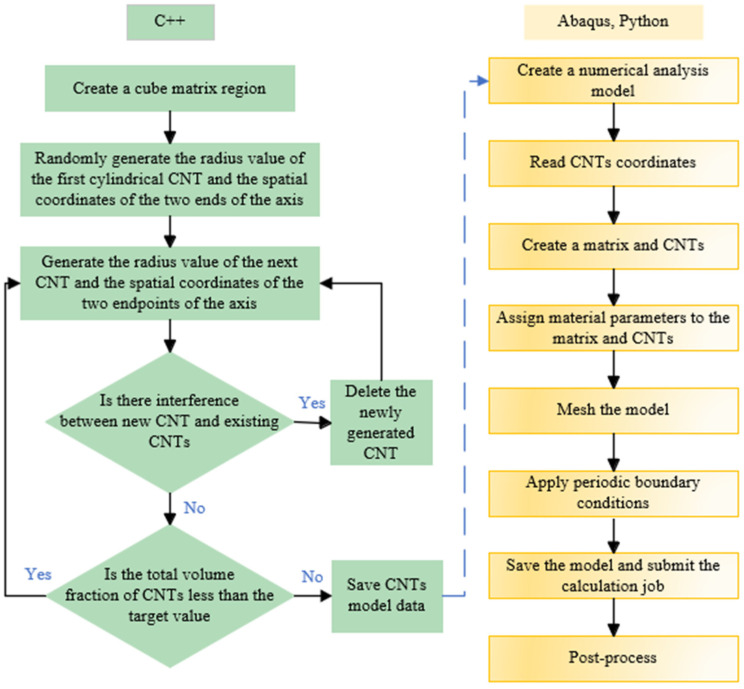
The flowchart of the modeling process.

The complete modeling process of the RVE model involves utilizing commercial software Abaqus to conduct FE analysis and determine the mechanical properties of composites. The flowchart illustrating the modeling process is depicted in Figure 5.

## 3. Simulation

**Figure 6:**
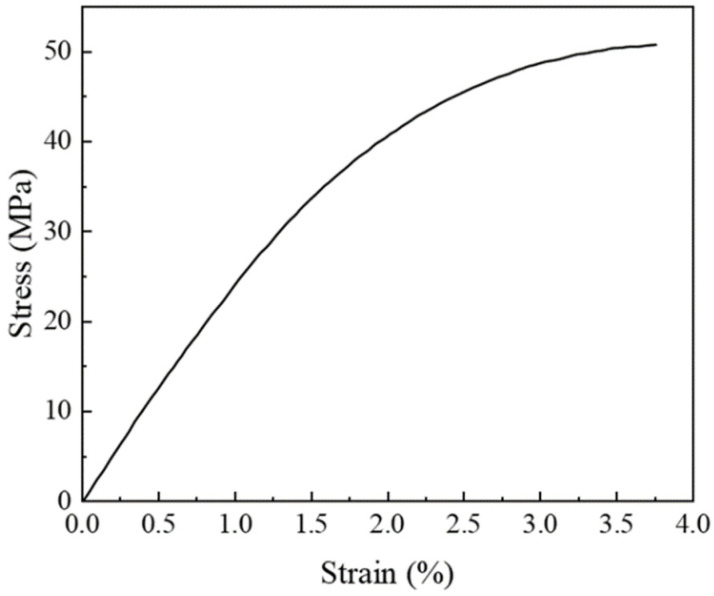
The stress–strain curve of epoxy resin [28].

To evaluate the effectiveness of the previously mentioned modeling approach, the RVE models of multi-walled CNTs embedded in epoxy resin composites with varying volume fractions of 1%, 1.5%, and 2% were developed. The simulation results of uniaxial tension were then compared with experimental data from the study cited in reference [28]. In the modeling process, CNTs were represented as equivalent cylinders and were assumed to be linear elastic isotropic materials [37,38,39]. The CNTs exhibited an average length of 5 μm and a diameter of 50 nm, resulting in an aspect ratio of 100. The material parameters employed in the simulation are presented in Table 1 [40,41,42]. Additionally, the stress–strain curve of the epoxy resin cited in reference [28] is depicted in Figure 6.

**Figure 7:**
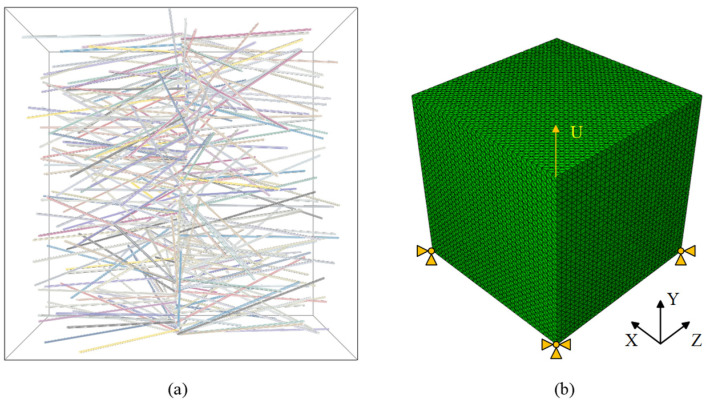
The 3D RVE model of CNTs filled epoxy resin is illustrated in two parts: (**a**) geometry and (**b**) meshed configuration.

Utilizing the commercial finite element software Abaqus, an RVE model of CNTs filled epoxy resin was created for uniaxial tensile simulation. The model dimensions are 10 μm × 10 μm × 10 μm, and it was subjected to displacement loading. The CNTs were discretized using C3D8R reduced integral elements with enhanced hourglass control, whereas the epoxy resin was represented by C3D4 elements. Considering the precision required for calculations, the scale, and associated costs, the configuration of the CNT axial grid is set to 20, while the circumferential grid is established at 12. The resin grid size is specified as 0.5 μm, resulting in a total grid count of approximately 1 million. The anticipated computation time utilizing an Intel^®^ Core^TM^ i7-10700 processor (Intel, Santa Clara, CA, USA) is estimated to be around 5 h. The boundary conditions and mesh division of the model are illustrated in Figure 7.

The macroscopic stress–strain curve of CNTs filled epoxy resin cannot be directly determined through FE analysis. However, it can be indirectly derived from the stress and strain of the elements using the volume averaging method [43]. The volume averaging method is a homogenization model that allows for the expression of average stress and strain as:

(11) $$\begin{array}{l} \bar{\sigma }=\frac{\sum_{i=1}^{N}\sigma_{i}}{\sum_{i=1}^{N}V_{i}}\Rightarrow \bar{\sigma }=\frac{1}{Vol}\int_{V}\sigma \partial V\\ \bar{\varepsilon }=\frac{\sum_{i=1}^{N}\varepsilon_{i}}{\sum_{i=1}^{N}V_{i}}\Rightarrow \bar{\varepsilon }=\frac{1}{Vol}\int_{V}\varepsilon \partial V \end{array}$$

In the given context, *Vol* denotes the cumulative volume of all elements within the RVE model, where *i* and *N* refer to the quantity and total number of elements, respectively. The variables $\bar{\sigma }$ and $\bar{\varepsilon }$ represent the average stress and strain of the model. Consequently, five randomly generated RVE models were examined using the FE method. By utilizing the provided equation and the stress and strain values extracted from the models, it is possible to determine the relationship between stress and strain.

## 4. Results and Discussion

**Figure 8:**
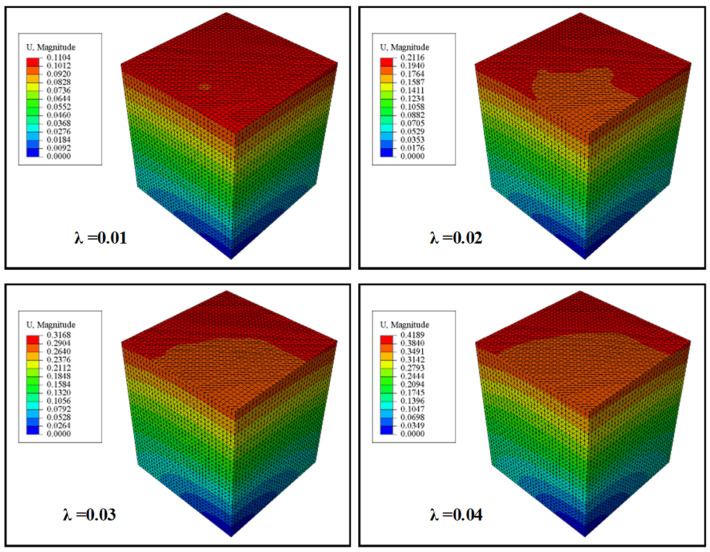
The displacement distribution of a 3D RVE model of an epoxy resin composite filled with 1.5% volume fraction of CNTs is analyzed under various uniaxial elongation ratios (λ).

By considering the CNTs filled epoxy resin composites with a volume fraction of 1.5%, the FE analysis outcomes of the RVE model are depicted in Figure 8. The analysis reveals that the matrix experiences gradual elongation in the loading direction while being compressed in the perpendicular directions as the elongation progresses.

**Figure 9:**
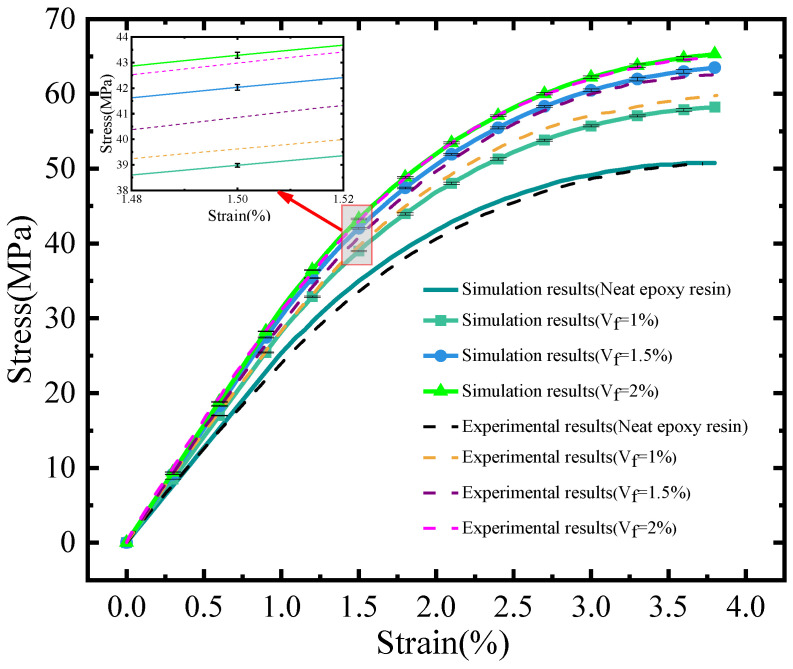
A comparison of simulation results with experimental data.

The simulation results for various volume fractions are presented in Figure 9. These results were derived from averaging the FE analysis outcomes of five randomly generated RVE models. The simulation results for pure resin are consistent with experimental data concerning general trends and qualitative evaluations. This indicates that, notwithstanding certain inaccuracies, the simulation remains proficient in predicting material behavior. The standard deviations of the simulation outcomes for the three different volume fractions are all below 0.3 MPa, which implies stability, minimal dispersion, and a high level of reliability in the simulation results. The stress demonstrated nonlinear variation as the strain increased. When the strain was below 1%, the stress–strain curve exhibited an approximately linear upward trend. Subsequently, the curve’s slope gradually decreased, indicating the entry of the epoxy resin into the plastic deformation stage, as depicted in Figure 9. Moreover, an increase in the volume fraction of CNTs led to a significant rise in the elastic modulus of the CNTs filled epoxy resin composite.

In comparison to the data presented in reference [28], the simulation results for three distinct volume fractions exhibited a consistent trend with the experimental findings; however, notable discrepancies were observed between the values, particularly during the plastic deformation stage (ε≥1%). It was noted that as the volume fraction increased, the error tended to diminish. This phenomenon can be attributed to the modeling process, wherein a higher filling amount facilitates a more uniform distribution of CNTs, thereby aligning more closely with the properties of the actual material [44]. Meanwhile, the agglomeration resulting from a high filling content significantly diminishes the strength enhancement capacity of CNTs, which in turn leads to a reduction in the mechanical properties of the composites, including Young’s modulus and tensile strength [45]. As a result, the simulation outcomes are marginally higher than the experimental data; however, this discrepancy is not considerable.

## 5. Conclusions

To accurately represent the random distribution of CNTs within a matrix, a 3D RVE modeling approach was developed to simulate the mechanical properties of CNTs filled polymers. This method utilizes the modified NNA and an interference judgment technique to generate the spatial coordinate distribution of CNTs within the RVE model. The modified NNA algorithm demonstrated superior randomness in generating CNTs coordinates compared to the RSA algorithm, aligning more closely with the actual distribution within the matrix. Subsequently, a 3D RVE model of CNTs filled polymer was constructed using Abaqus software. Through this modeling technique, the uniaxial tensile mechanical response of CNTs filled epoxy resin was analyzed. The stress–strain curve of the CNTs filled composite was obtained by averaging the stress and strain results of the finite elements obtained from the FE analysis.

The validity of the RVE modeling approach was verified by comparing the simulation outcomes for various volume fractions with experimental data from the existing literature. Despite the discrepancies observed between the simulation and experimental results, particularly during the phase of plastic deformation attributed to model simplifications and assumptions, the proposed method demonstrates efficacy in predicting the impact of microstructural parameters, such as filling ratios and distribution patterns, on the performance of CNTs filled polymers. Furthermore, the integration of the modified NNA algorithm with the interference judgment method introduced in this study enhances both the randomness and aspect ratio of the generated CNTs, while concurrently ensuring a high filling volume. This approach facilitates a distribution of CNTs within the RVE model that more accurately reflects actual filling conditions, thereby expanding its applicability. Such advancements are crucial for elucidating the physical and mechanical properties of the CNTs filled polymers, thereby enhancing material design and improving the performance of composites in practical applications. Additionally, this methodology offers a theoretical framework for the development of novel materials, while simultaneously reducing experimental costs and time.

## Figures and Tables

**Table 1:** The material parameters of epoxy resin and CNTs [40,41,42].

Material	Young’s Modulus (GPa)	Poisson’s Ratio	Tensile Strength (GPa)
Epoxy resin	2.538	0.3	0.0509
CNTs	260	0.17	20

## Data Availability

The data presented in this study are available on request from the corresponding author.

## Appendix A. Comparison of Interference Judgment Algorithms

The algorithm presented in references [16,17,18,19] is designed to ascertain the overlap between two cylinders by reducing each cylinder to a line segment. Specifically, these algorithms initiate the process by calculating the distances between the four endpoints of the two line segments that represent the cylinders. Subsequently, they determine the distance from the endpoint of one line segment to the line defined by the other line segment. Finally, the length of the common perpendicular line between the two line segments is computed. This algorithm evaluates whether the shortest distance between the line segments is less than a predetermined threshold, which serves as an indicator of potential overlap between the cylinders.

Nevertheless, this method exhibits certain limitations. While it is capable of recognizing all overlapping cylinders, it may erroneously classify some non-overlapping cylinders as overlapping. To address this issue, the algorithm proposed in this article incorporates several significant enhancements. It categorizes the spatial distribution of line segments into six distinct scenarios, as illustrated in Figure 3. By assessing the spatial relationships of cylinders within these specific contexts, the improved algorithm mitigates the misclassification errors that were present in the original method.

To illustrate the superiority of the enhanced algorithm, we present a comparison in Table A1. This table delineates various scenarios that correspond to the cases labeled a–f in Figure 3. The columns A–D in the table provide the spatial coordinates of the four endpoints of the line segment that represents the cylinder. The value of d1 denotes the shortest distance between line segments as calculated by existing algorithms in the literature. The value of d2 represents the shortest distance determined by the enhanced algorithm proposed in this study. The value of d3 indicates the actual shortest distance between the two line segments based on the true geometric properties of a cylinder. The findings unequivocally demonstrate that the original algorithm exhibits accuracy solely in cases a and d; conversely, in all other instances, its outputs fall short of the true values. In contrast, the interference detection algorithm proposed in this article yields results that align closely with the actual values.

**Table A1:** An example display of interference judgment.

	A	B	C	D	d1	d2	d3
a	(0,0,0)	(0,2,0)	(1,1,0)	(−1,1,0)	0	0	0
b	(0,0,0)	(1,3,0)	(2,3,0)	(3,0,0)	0	1	1
c	(0,0,0)	(0,3,0)	(1,2,0)	(3,0,0)	0	1	1
d	(0,0,0)	(0,2,0)	(1,1,1)	(−1,1,1)	1	1	1
e	(0,0,0)	(1,0,0)	(2,1,1)	(3,2,1)	1	$\sqrt{3}$	$\sqrt{3}$
f	(0,0,0)	(2,0,0)	(0,2,1)	(1,1,1)	1	$\sqrt{2}$	$\sqrt{2}$
